# Hydroxychloroquine blocks SARS-CoV-2 entry into the endocytic pathway in mammalian cell culture

**DOI:** 10.1038/s42003-022-03841-8

**Published:** 2022-09-14

**Authors:** Zixuan Yuan, Mahmud Arif Pavel, Hao Wang, Jerome C. Kwachukwu, Sonia Mediouni, Joseph Anthony Jablonski, Kendall W. Nettles, Chakravarthy B. Reddy, Susana T. Valente, Scott B. Hansen

**Affiliations:** 1 Department of Molecular Medicine, The Scripps Research Institute, Jupiter, FL 33458 USA; 2 Department of Neuroscience, The Scripps Research Institute, Jupiter, FL 33458 USA; 3 Skaggs Graduate School of Chemical and Biological Sciences, The Scripps Research Institute, Jupiter, FL 33458 USA; 4 Department of Integrative Structural and Computational Biology, The Scripps Research Institute, Jupiter, FL 33458 USA; 5 Department of Immunology and Microbiology, The Scripps Research Institute, Jupiter, FL 33458 USA; 6 Department of Internal Medicine, University of Utah Health Sciences Center, Salt Lake City, UT 84112 USA

**Keywords:** SARS-CoV-2, Nanoscale biophysics, Membrane trafficking, Membrane lipids, Sterols

## Abstract

Hydroxychloroquine (HCQ), a drug used to treat lupus and malaria, was proposed as a treatment for SARS-coronavirus-2 (SARS-CoV-2) infection, albeit with controversy. In vitro, HCQ effectively inhibits viral entry, but its use in the clinic has been hampered by conflicting results. A better understanding of HCQ’s mechanism of actions in vitro is needed. Recently, anesthetics were shown to disrupt ordered clusters of monosialotetrahexosylganglioside1 (GM1) lipid. These same lipid clusters recruit the SARS-CoV-2 surface receptor angiotensin converting enzyme 2 (ACE2) to endocytic lipids, away from phosphatidylinositol 4,5 bisphosphate (PIP_2_) clusters. Here we employed super-resolution imaging of cultured mammalian cells (VeroE6, A549, H1793, and HEK293T) to show HCQ directly perturbs clustering of ACE2 receptor with both endocytic lipids and PIP_2_ clusters. In elevated (high) cholesterol, HCQ moves ACE2 nanoscopic distances away from endocytic lipids. In cells with resting (low) cholesterol, ACE2 primarily associates with PIP_2_ clusters, and HCQ moves ACE2 away from PIP_2_ clusters—erythromycin has a similar effect. We conclude HCQ inhibits viral entry through two distinct mechanisms in high and low tissue cholesterol and does so prior to inhibiting cathepsin-L. HCQ clinical trials and animal studies will need to account for tissue cholesterol levels when evaluating dosing and efficacy.

## Abstract (web summary)

Super-resolution microscopy in cultured cells is employed to dissect the effect of hydroxychloroquine (HCQ) at the plasma membrane and HCQ directly perturbs clustering of the SARS-CoV-2 receptor ACE2 with endocytic lipids and PIP2 clusters.

## Introduction

Coronavirus disease 2019 (COVID-19), a viral infection caused by severe acute respiratory syndrome coronavirus 2 (SARS-CoV-2), recently emerged as a serious public health problem^1,2^. Currently, millions of people have been infected with SARS-CoV-2 worldwide. Proposed treatments for severe symptoms include a well-known FDA-approved antimalarial and anti-inflammatory agents chloroquine (CQ) and its derivative hydroxychloroquine (HCQ)^3–7^, but their mechanisms of action are poorly understood in human cells (and in particular in the presence of underlying conditions). A retrospective study claimed a benefit in particular with the macrolide antibiotic azithromycin^8^. However, their use is not without controversy^9,10^, and randomized control studies without an antibiotic appeared to have no benefit^11^. In the treatment of malaria, CQ targets the replication cycle of the parasite^12^; This mechanism of action is presumably unrelated to their actions in COVID-19, Lupus, and Niemann-Pick Syndrome. Understanding the underlying in vitro mechanism for these compounds in COVID-19 could help in understanding their mechanism of action in humans, in designing efficacious clinical trials, and in bettering the translation of their use in the clinic.

Previous research from the lab shows that a cholesterol-dependent mechanism for anesthetics regulates the movement of membrane proteins between monosialotetrahexosylganglioside1 (GM1) lipid clusters and PIP_2_ lipid clusters through perturbing the affinity of proteins for GM1 clusters^13,14^. While the GM1 clusters are formed by cholesterol packing (Van der Waals interactions) with palmitates covalently attached to proteins (palmitoylation)^15^, the PIP_2_ clusters are formed from charged-protein clustering^16^ (Supplementary Fig. 1a) located near disordered lipids. We refer to the nonclustered region as the ‘disordered region’ of the cell since it is separate from GM1 clusters^17^ and contains unsaturated lipids that are disordered. PIP_2_ is thought to cluster near disordered lipids^18,19^. In cellular membranes, both local and general anesthetics act as a chaotrope to disrupt the cholesterol-induced clustering of palmitoylated proteins^13,20^. Within this process, cholesterol protects against the disruption of cells by sealing the air-water interface of the lipid membranes^21^.

Cholesterol is critical to both viral entry and immune responses^22^. The cholesterol-rich GM1 clusters facilitate endocytosis^23,24^. SARS-CoV-2 surface receptor (angiotensinogen converting enzyme 2 (ACE2))^25–27^ has recently been shown to move between GM1 clusters and PIP_2_ clusters in a cholesterol-dependent manner^28^. As the cellular cholesterol level rises, both the number of endocytic lipids and their apparent cluster size increase. In an obese mouse model, cholesterol was high in lung tissue, and this correlated with ACE2 movement to the endocytic lipids, a condition that accelerated viral entry into the target cells in cell culture^28^.

Cholesterol in blood appears to be low in multiple studies of COVID-19 patients with severe disease^29,30^. However, the cellular cholesterol concentration measured in monocytes of the same patients was elevated^30^, suggesting an opposite result in blood and tissue at the late stages of the disease. Importantly, cholesterol synthesis and uptake into immune cells is a key effector of inflammation^31^. The cholesterol transport protein apolipoprotein E (apoE) both loads and unloads cholesterol to and from cellular membranes^32^, allowing us to manipulate cholesterol levels both in vitro and in vivo (Supplementary Fig. 1b).

**Fig. 1:**
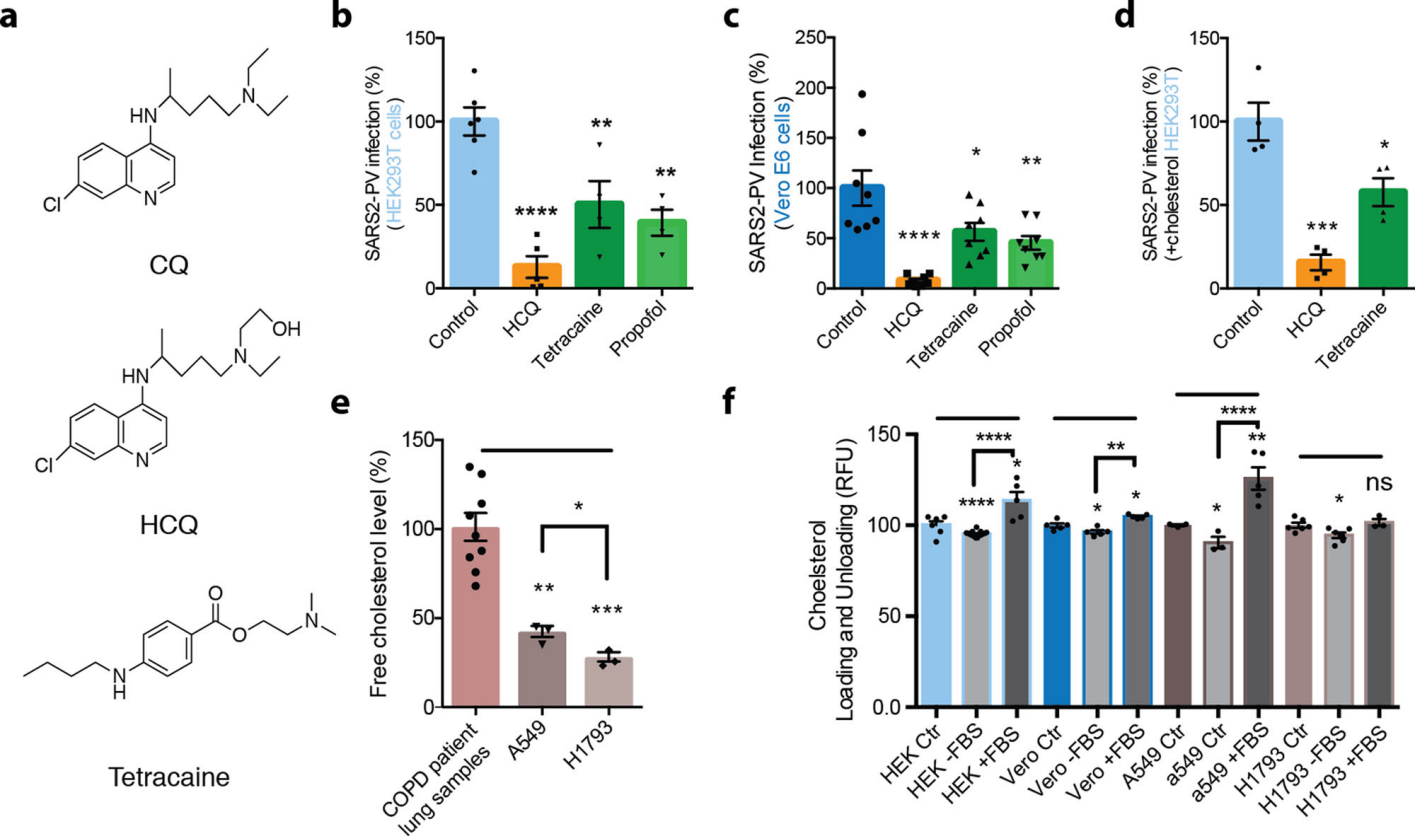
Anesthetics and hydroxychloroquine inhibit SARS2-PV entry into cultured cells. **a** Chemical structure comparing chloroquine (CQ) and hydroxychloroquine (HCQ) to tetracaine, a local anesthetic. **b**–**d** SARS-CoV-2 pseudovirus (SARS2-PV) entry assay measured as a percent of control luciferase activity. HCQ (50 μM), tetracaine (50 μM), and propofol (50 μM) inhibited viral infection in wt. Vero E6 cells (**c**) and ACE2 overexpressing HEK293T cells without (**b**) and with (**d**) cholesterol loading (4 μM apolipoprotein E + 10% serum). Data are expressed as mean ± s.e.m., *P ≤ 0.05, **P ≤ 0.01, ***P ≤ 0.001, ****P ≤ 0.0001, one-way ANOVA with post hoc Tukey’s test, *n* = 4–8. **e** Free-cholesterol level of airway lung tissue from patients with chronic obstructive pulmonary disease, A549 cells and H1793 cells indicated by raw resorufin signals readout. Data are expressed as mean ± s.e.m., **P ≤ 0.01, ***P ≤ 0.001, one-way ANOVA with post hoc Tukey’s test, *n* = 3. Free-cholesterol level of A549 cells and H1793 cells are compared using unpaired *t*-test, *P ≤ 0.05. **f** Loading and unloading of cholesterol with apolipoprotein E (apoE) in the presence (+FBS) and absence (−FBS) of fetal bovine serum (FBS) as a source of cholesterol. All cells used for the cholesterol assay are wild-type: kidney cell lines HEK293T (HEK) and Vero E6 (vero) and lung cell lines A549 and H1793. Data are expressed as mean ± s.e.m., *P ≤ 0.05, **P ≤ 0.01, ***P ≤ 0.001, ****P ≤ 0.0001, ns not significant, unpaired *t*-test.

Interestingly, CQ is an anesthetic—subcutaneous injections of CQ produce instant local anesthesia sufficient to perform a surgical procedure^33,34^. Also, both CQ and tetracaine, a local anesthetic, are hydrophobic and contain a tertiary amine (Fig. 1a). Since CQ and local anesthetics (such as tetracaine) are weak bases, their uptake changes the acid-base balance within the membrane^35,36^. Additionally, common local anesthetics (such as mepivacaine, bupivacaine, and tetracaine) and other GM1 cluster disrupting compounds (such as sterols and cyclodextrin) can exert antiviral or antimicrobial activity^37–40^. Terpenoids can also disrupt viral entry; this process is cholesterol-dependent^41^.

Both the cholesterol-dependent lipid disruption and antiviral properties of anesthetics, along with the anesthetic and antiviral properties of HCQ link the lipid disruption properties of HCQ with its viral-entry inhibiting effects. Further research into this link could help us understand both HCQ and anesthetics’ underlying molecular mechanism(s) in mammalian cells; in particular, mammalian cells in a high-cholesterol state—a state that is consistent with the chronic inflammation and the comorbidities of COVID-19^42^, lupus^43,44^, and Niemann pick^45^. Prior models speculated that HCQ inhibited cathepsin-L by changing the endosomal pH^46^. However, if HCQ disrupts ACE2 clustering, it would reverse the effects of cholesterol and inhibit the endocytic entry prior to the cathepsin-L cleavage. Here we use super-resolution imaging to show that HCQ disrupts the clustering of ACE2 with both endocytic lipids and PIP_2_.

## Results

### Inhibition of SARS-CoV-2 entry by anesthetic compounds

In order to test a membrane-disruptive mechanism for HCQ inhibition of SARS-CoV-2 viral entry, we compared HCQ to anesthetics (tetracaine and propofol) which are known to be membrane-disruptive. HEK293T cells overexpressing ACE2 were infected with a retrovirus pseudotyped with the SARS-CoV-2 spike protein (SARS2-PV). A segment of the spike protein binds to ACE2 and recapitulates viral entry^47,48^. A luciferase encoded in the pseudotyped virus is then used to quantitate viral entry (Fig. 1b–d).

Treatments with HCQ, tetracaine, and propofol all robustly reduced SARS2-PV entry into HEK293T cells overexpressing ACE2 (Fig. 1b). The cells were first treated with drugs (50 µM) for 1 h, then the drugs were removed. After the treatment and subsequent drug removal, SARS2-PV was applied such that the virus was never exposed to the drugs, thus avoiding potential direct effects of cholesterol on the viron. HCQ had the greatest effect on viral inhibition with almost a 90% reduction in SARS2-PV luciferase activity (Fig. 1b). We used 50 µM since that concentration was previously shown to be the minimum concentration that fully inhibited viral entry^3^. The concentration is ~5-fold above the concentration found in lung epithelial lining fluid after 400 mg for 1 day^49^ making it an appropriate concentration to see a full effect by dSTORM. Like anesthetics, the actual concentration of HCQ in the membrane is dictated by a partition coefficient and the resultant mole fraction, not external concentration.

In Vero E6 cells, a cell line that endogenously expresses ACE2 receptor and robustly facilitates SARS-CoV-2 viral entry^50^, HCQ, tetracaine, and propofol all significantly decreased viral entry (Fig. 1c). HCQ again showed the strongest effect decreasing viral entry by ~92%. Cell viability after HCQ treatment was assessed by Fixable Viability Dye (FVD) staining and MTT assay. The FVD staining labeled dead cells and found that HCQ treatment had no effect on Vero E6 cells, in agreement with a previous study^3^, but did decrease live cell number by ~23.93 ± 5% in HEK293T cells (Supplementary Fig. 1c, d). Similarly, the MTT assay showed HCQ treatment significantly decreased cell metabolism in HEK293T cells by ~36.05 ± 6% (Supplementary Fig. 1d). However, the reduction in cell viability of HCQ did not account for the full reduction in viral entry. Tetracaine and propofol had no adverse effects on cell viability in vero E6 or HEK293T cells (Supplementary Fig. 1e).

COVID-19 is often severe in obese patients and those with underlying conditions. We obtained lung samples from adult humans with chronic obstructive pulmonary disease (COPD). We found the lung tissue to have significantly higher free-cholesterol levels compared to cultured lung cell lines as measured by our fluorescent cholesterol assay (Fig. 1e). To recapitulate the physiological conditions observed in COVID-19 patients, we tested HCQ’s inhibition on viral entry in HEK293T loaded with cholesterol and overexpressing ACE2. To load cholesterol into cells, 4 µM apolipoprotein E (apoE, a cholesterol carrier protein linked to the severity of COVID-19^51^) was applied. ApoE binds to low-density lipoprotein (LDL) receptors in tissue and facilitates the loading of cholesterol into cells^52^ (Supplementary Fig. 1b). To provide a source of cholesterol to the apoE, 10% fetal bovine serum (FBS, a common source of cholesterol) totaling ~310 µg/mL was added. Importantly, apoE is not present in FBS, allowing us to carefully control cholesterol loading^32,53^. When apoE is in excess or in low-cholesterol conditions, it facilitates the efflux of cholesterol from the cell^32,52^. Cells were treated acutely (1 h) for loading or unloading cholesterol prior to viral infection.

Loading cells with cholesterol into HEK293T cells overexpressing ACE2 increased viral entry by ~56 ± 38% (Supplementary Fig. 1f), which is consistent with observations with endogenously expressed ACE2 where cholesterol loading significantly increased viral entry by ~36 ± 7% (Supplementary Fig. 1g). As expected, treatment of cholesterol loaded cells with HCQ (~85 ± 12%) and tetracaine (~43 ± 12%) reduced SARS2-PV entry in a high-cholesterol state (Fig. 1d). The efficacy of HCQ was reduced in cholesterol loaded cells compared to non-cholesterol loaded cells, but only slightly.

To confirm that apoE loads and unloads cholesterol from cultured cells, we treated HEK293T, Vero E6, and A549 cells with apoE with and without 10% FBS and measured the relative change in membrane-free cholesterol (Fig. 1f). Cells that were incubated with and without a source of cholesterol contained small but significant increases and decreases in total cholesterol respectively. The loading and unloading of cholesterol were similar in H1793 cells, although the loading of cholesterol did not reach statistical significance.

### HCQ’s disruption of ordered GM1 clusters

**Fig. 2:**
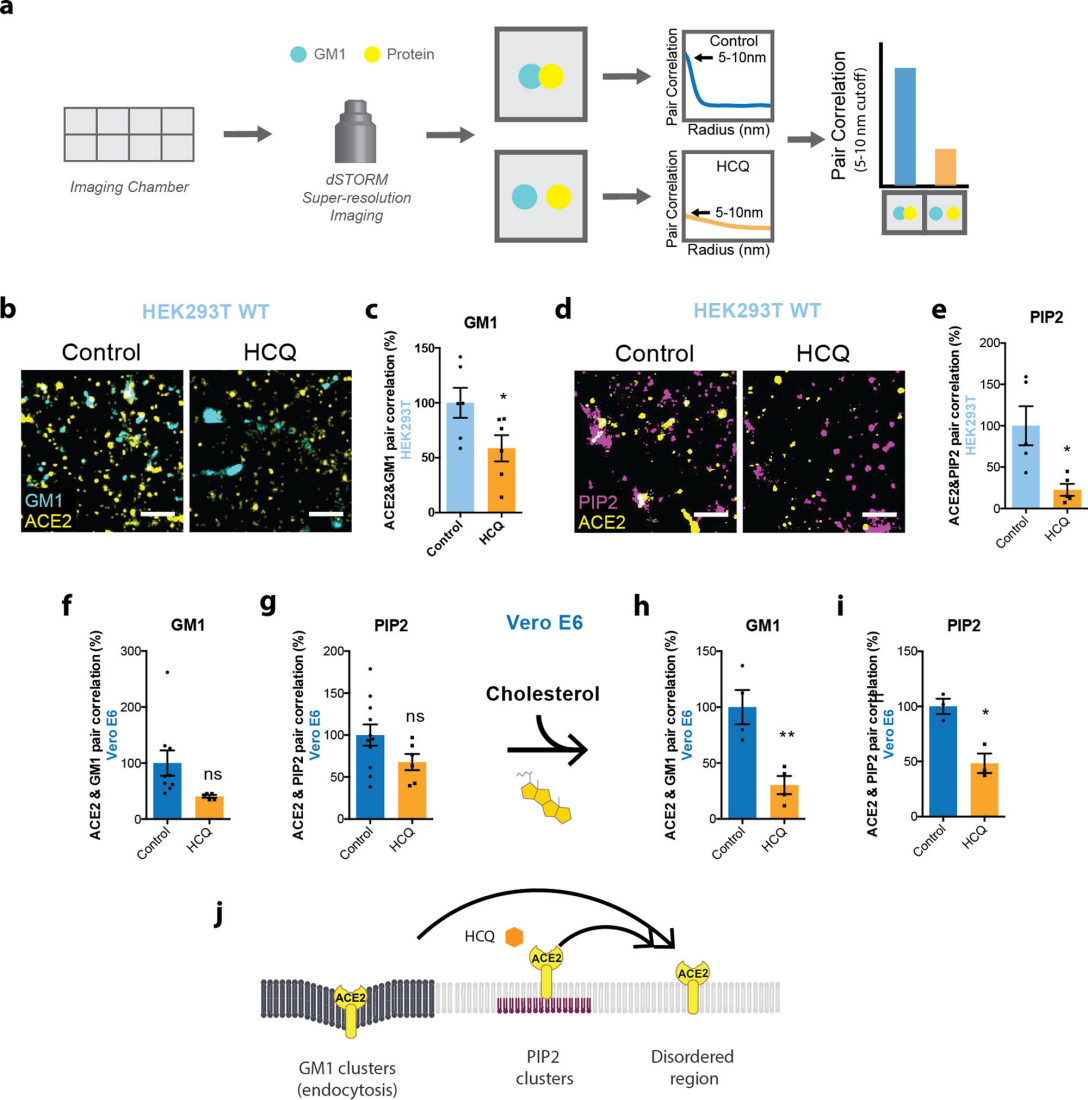
Hydroxychloroquine displaces ACE2 from both GM1 and PIP2 clusters in kidney cells. **a** Representative figure showing the process of dSTORM super-resolution imaging. Cells were cultured in eight-well imaging chambers and imaged with the super-resolution microscope. Pair correlation was analyzed to determine colocalization of two probes. **b** Representative dSTORM super-resolution images showing the effect of HCQ (50 μM) on the nanoscale localization of ACE2 (yellow) with GM1 clusters (cyan) after loading HEK293T cells with cholesterol (scale bars = 1 μm). **c** Percent of pair correlation (Supplementary Fig. 3a) calculated at short distances (0–5 nm). HCQ decreased the pair correlation between ACE2 and GM1 clusters indicating a decrease in association between PLD and GM1 clusters. Data are expressed as mean ± s.e.m., *P ≤ 0.05, unpaired *t*-test, *n* = 6. **d** Representative dSTORM super-resolution images of ACE2 (yellow) and PIP_2_ cluster (magenta) in HEK293T cells at normal cholesterol level after the treatment of HCQ (50 μM) (scale bars = 1 μm). **e** Percent of pair correlation (Supplementary Fig. 3b) calculated at short distances (0–5 nm). HCQ decreased the pair correlation between ACE2 and PIP_2_ clusters indicating a decrease in association between PLD and PIP_2_ clusters. Data are expressed as mean ± s.e.m., *P ≤ 0.05, unpaired *t*-test, *n* = 5. **f**, **g** HCQ trended to a decrease in the pair correlation between ACE2 and GM1 clusters (**f**) and ACE2 and PIP_2_ clusters (**g**) at resting cholesterol levels in Vero E6 cells without statistical significance. Data are expressed as mean ± s.e.m., *P* = 0.0789, unpaired *t*-test, *n* = 5–9 for GM1 staining and *P* = 0.1071, unpaired *t*-test, *n* = 6–11 for PIP_2_ staining; ns not significant. **h, i** HCQ decreased the pair correlation between ACE2 and GM1 clusters (**h**) and ACE2 and PIP2 clusters (**i**) after cholesterol uptake with apolipoprotein E in Vero E6 cells. Data are expressed as mean ± s.e.m., **P ≤ 0.01, *P ≤ 0.05; *n* = 3–4 unpaired *t*-test. **j** Model showing HCQ (orange hexagon) inducing translocation of ACE2 (yellow receptor) from GM1 clusters (dark gray lipids) and PIP_2_ clusters (purple lipids) to disordered region (light gray lipids) in HEK293T cells and Vero E6 cells loaded with cholesterol.

The ability of a virus to cluster is important for its infectivity and maturation^54–57^. Previously, anesthetics were shown to perturb clustering in two ways. First, inhaled anesthetics tend to increase the apparent size and number of clusters, as observed using super-resolution imaging and cluster analysis, while local anesthetics tend to decrease the cluster size^13,20^. Second, both inhaled and local anesthetics disassociate cholesterol-sensitive proteins from GM1 clusters. The dissociation of proteins from a GM1 cluster is recorded by two-color direct stochastic optical reconstruction microscopy (dSTORM) super-resolution imaging. The GM1 and PIP_2_ lipid, and ACE2 protein are fixed and labeled with cholera toxin B (CTxB, a pentadentate toxin binding GM1 lipids), PIP_2_ antibody, and ACE2 antibody, respectively, and then ACE2 association with the lipid is measured by pair correlation analysis (Fig. 2a). The antibodies in this study were previously validated for specificity (see methods). We previously used these techniques to monitor nanoscopic movement (<100 nm) of multiple proteins between both GM1 and PIP_2_ clusters^13,20,28,32,58^ (see also Discussion).

To test HCQ’s effects on lipid membranes, the effect of HCQ was first examined on the apparent structure (size and number) of GM1 clusters by dSTORM in the membranes of HEK293T cells. This was done using density-based spatial clustering of applications with noise (DBSCAN). The use of HEK293T cells allowed us to compare the effects of HCQ to previous anesthetic studies in HEK293T cells^13,20^. As mentioned, 50 µM HCQ is the minimum saturating concentration that was shown to inhibit viral entry in cultured cells^3^.

We tested 50 µM HCQ and found it increased the number and apparent size (Supplementary Fig. 2a–c) of GM1 clusters, despite lowering the free cholesterol in the membrane (Supplementary Fig. 1h). HCQ’s perturbation to cluster size in HEK293T cells was most similar to the inhaled anesthetics chloroform and isoflurane^13^ (Supplementary Fig. 2a). Methyl-beta cyclodextrin (MβCD), a chemical known to deplete GM1 clusters from the cell membrane, decreased the apparent size of GM1 clusters and showed a trend of decreasing cluster numbers (Supplementary Fig. 2b, c). Interestingly, HCQ had the opposite effect on lung cells. HCQ decreased cluster size and a number of GM1 clusters, a result that is similar to what was seen for local anesthetics in nerve cells (Supplementary Fig. 2d–f)^20^.

### HCQ’s effects on ACE2 clustering in kidney and lung cells

In cells and animals with low cholesterol, ACE2 clusters primarily with PIP_2_; however, in high-cholesterol and obese animals, ACE2 appears to cluster primarily with GM1 lipids^28^. To compare the effect of HCQ on ACE2 clustering in conditions of high and low cholesterol, we loaded and unloaded cholesterol into and from wild-type (wt.) HEK293T and Vero E6 cells (kidney) with apoE in the presence and absence of serum cholesterol. The cells were then fixed, labeled with anti-ACE2 antibody and CTxB, and imaged using two-color dSTORM. Both cell lines express low levels of endogenous ACE2^32,59^.

We found a 50 µM HCQ treatment in HEK293T cells dramatically decreased ACE2 receptor association with GM1 clusters, despite increases in both GM1 cluster size and number (Supplementary Fig. 2a–c). Figure 2b shows representative dSTORM images comparing the disruption of HCQ on colocalization of ACE2 and GM1 clusters. At short distances (0–10 nm), pair correlation decreased 41 ± 18% (*p* < 0.05) (Fig. 2c, S3A), confirming that HCQ acts as a chaotrope to disrupt the ability of GM1 clusters to sequester ACE2. This result is in agreement with its anesthetic-like mechanism of action and its effect on PLD2^13^.

As mentioned earlier, ACE2 moves to PIP_2_ clusters in resting/low-cholesterol conditions. PIP_2_ clusters reside near disordered lipids apart from GM1 clusters due to a large amount of unsaturation in PIP_2_’s acyl chains^18,60^. To determine whether ACE2 moves to PIP_2_ clusters after HCQ’s disruption of GM1 clusters, ACE2 and PIP_2_ clusters were co-labeled in HEK293T cells at resting/low-cholesterol levels, then treated with/without 50 µM HCQ.

Figure 2d shows representative dSTORM images comparing PIP_2_ clusters (purple labeling) before/after HCQ treatment in HEK293T cells. Surprisingly, the pair correlation between ACE2 and PIP_2_ clusters was decreased (Fig. 2e) at all distances (Supplementary Fig. 3b). The decrease of 77 ± 25% at short distances (0–10 nm) (*p* < 0.05) suggests that HCQ disrupts ACE2 association with both GM1 clusters and PIP_2_ clusters, presumably forcing the protein to disordered lipids in the plasma membrane. Further characterization of the PIP_2_ clusters showed HCQ treatment decreased both the size and number of PIP_2_ clusters by 20 ± 4%, and 44 ± 13% respectively (Supplementary Fig. 3c, d).

Next, we tested Vero E6 cells, which, as mentioned, endogenously express ACE2. Probing the nanoscale trafficking of ACE2 with endogenously expressed protein is important as overexpression can overwhelm the ability of GM1 lipids to sequester the receptor away from PIP_2_ and alter imaging results. Cholesterol-treated cells were fixed and stained for ACE2, and either GM1 clusters or PIP_2_ clusters. Within low-cholesterol conditions, ACE2 colocalized to both GM1 and PIP_2_ clusters (Supplementary Fig. 4a, b, Supplementary Table 1). Fifty micromolar HCQ treatment of ACE2 reduced the pair correlation between ACE2 and both GM1 clusters (59 ± 31%, *p* = 0.0789) and PIP_2_ clusters (32 ± 19%, *p* = 0.1071) (Fig. 2f, g).

In Vero E6 cells with elevated cholesterol, again, ACE2 associated with both GM1 and PIP_2_ clusters. However, 50 μM HCQ treatment had the greatest effect on ACE2’s association with both clusters (70 ± 17%, *p* < 0.01) (Fig. 2h, Supplementary Fig. 2g) compared to PIP_2_ clusters (52 ± 11%, *p* < 0.05) (Fig. 2i, Supplementary Fig. 2h). This suggests HCQ has its greatest effect on endocytic lipids when cholesterol is high (Fig. 2j).

**Fig. 3:**
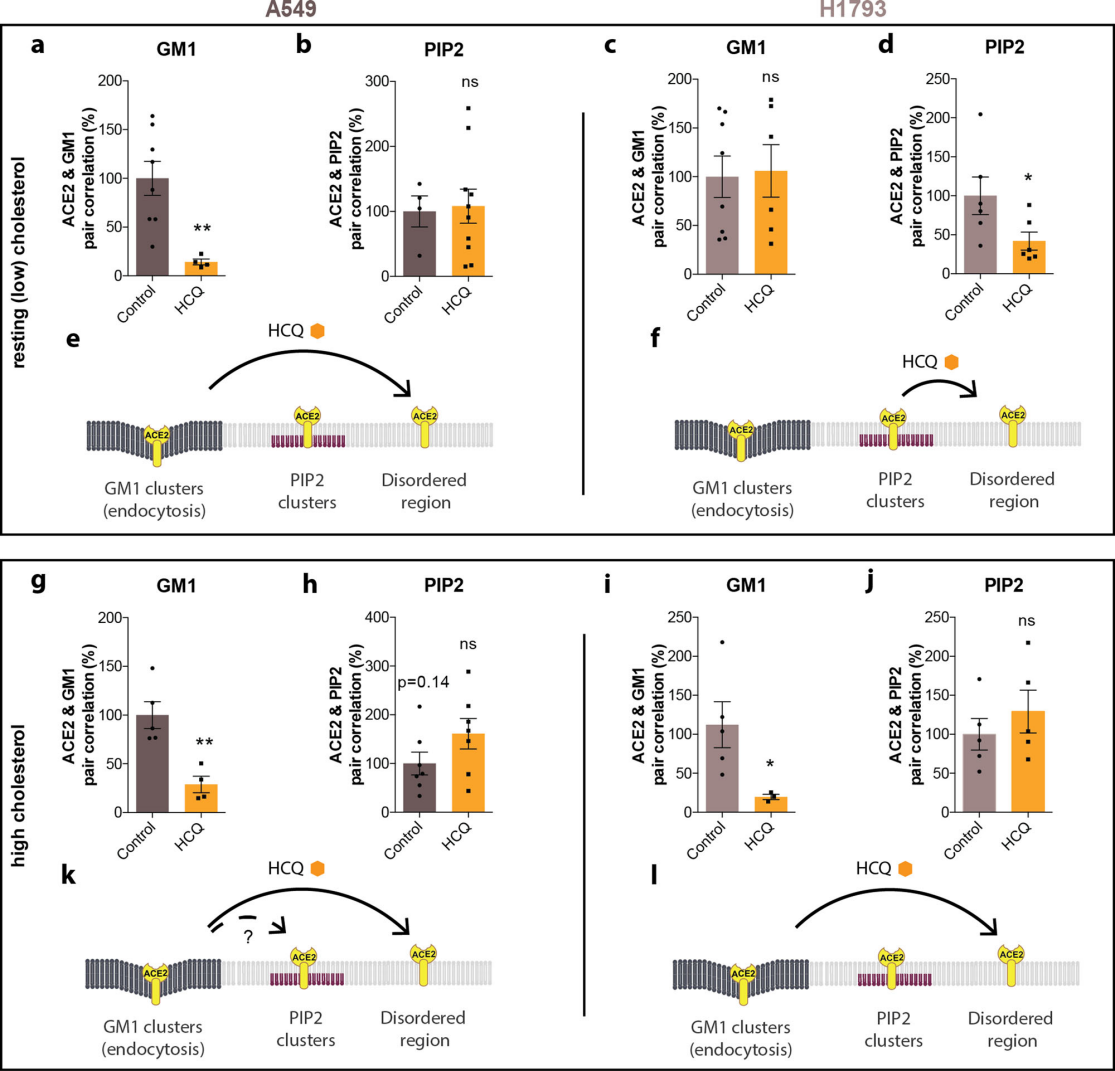
Cholesterol dictates hydroxychloroquine’s effect in lung cells. Top panels**:** Analysis at resting (low) cholesterol. **a**, **b** HCQ decreased the pair correlation between ACE2 and GM1 clusters at resting (low) cholesterol levels in A549 cells, but not ACE2 and PIP_2_ cluster, Values are graphed as mean ± s.e.m., **P ≤ 0.01, ns *P* = 0.8603 for PIP_2_. Unpaired *t*-test, *n* = 4–10. **c**, **d** In H1793 cells at the resting cholesterol level, HCQ had no significant effects on the pair correlation between ACE2 and GM1 clusters; ns *P* = 0.8627, unpaired *t*-test, *n* = 6–8; however, HCQ did significantly decrease pair correlation between ACE2 and PIP_2_ clusters. Data are expressed as mean ± s.e.m., **P ≤ 0.05, unpaired *t*-test, *n* = 6. **e** Model showing HCQ inducing translocation of ACE2 from GM1 clusters (dark gray lipids) to disordered region in cells with resting cholesterol. **f** Model showing HCQ inducing translocation of ACE2 from PIP_2_ clusters to disordered region in H1793 cells with resting cholesterol levels. Bottom Panels: Analysis after uptake of cholesterol using apoE treatment (high cholesterol). **g** In A549 with high cholesterol, HCQ decreased the pair correlation between ACE2 and GM1 clusters and **h** trended towards higher PIP_2_ correlation without significance *P* = 0.1435. Data are expressed as mean ± s.e.m., **P ≤ 0.01, unpaired *t*-test, *n* = 7. **i**, **j** In H1793 cells with high cholesterol, HCQ decreased the pair correlation between ACE2 and GM1 clusters but (**j**) had no significant effect on ACE2/PIP_2_ clustering (ns P = 0.4173). Data are expressed as mean ± s.e.m., *P ≤ 0.05, unpaired *t*-test, *n* = 4–6. **k** Model showing HCQ inducing translocation of ACE2 from GM1 clusters to PIP_2_ clusters and disordered region in cholesterol loaded A549 cells. **l** Model showing HCQ inducing translocation of ACE2 from GM1 clusters to PIP_2_ clusters and disordered region in cholesterol loaded H1793 cells.

Next, we tested A549 lung cells since HCQ appeared to affect the size of GM1 lipids differently compared to HEK293T and Vero (Supplementary Fig. 2). A549 lung cells were loaded in a manner identical to VeroE6 cells using apoE. Prior to HCQ treatment, ACE2 predominately associated with GM1 clusters in both resting cholesterol and high cholesterol. However, after loading cholesterol, ACE2 shifted out of PIP_2_ clusters (Supplementary Fig. 4c, d, i, j; Supplementary Table 1). Treatment with 50 μM HCQ disrupted colocalization of ACE2 receptors with GM1 clusters in both resting and high-cholesterol-treated cells (Fig. 3a, g), but not PIP_2_ (Fig. 3b, h). A trend toward increased PIP_2_ pair correlation suggested some translocation of ACE2 from GM1 to PIP_2_ clusters with high cholesterol may occur (*p* = 0.14, dashed arrow). The movement of ACE2 in A549 cells is summarized in Fig. 3e, k.

Lastly, we compared pair correlation in H1793 lung cells before and after cholesterol treatment. In resting H1793 cells (low cholesterol), most of the ACE2 was associated with PIP_2_ clusters (Supplementary Fig. 4e, f), but the association shifted toward GM1 clusters after loading cells with cholesterol (Supplementary Fig. 4k, l, Supplementary Table 1). After treatment with 50 μM HCQ, ACE2 was displaced from PIP_2_ clusters in resting cells and from GM1 clusters in high cholesterol (Fig. 3d, i), but not GM1 clusters in resting cholesterol or PIP_2_ clusters in high cholesterol (Fig. 3c, j). The movement of ACE2 in H1793 is summarized in Fig. 3f, l.

To estimate the distance ACE2 moves between compartments in the membrane, we labeled GM1 and PIP_2_ domains and performed two-color dSTORM on the lipids. We found the half maximal nearest neighbor ranges from 133 to 235 nm (Supplementary Table 1, and Supplementary Fig. 4s–v). The distribution of GM1 and PIP_2_ in the membrane appeared to be random, and their separation appeared uncorrelated with labeling intensity (Supplementary Fig. 4w, x).

### HCQ’s disruption of PLD2

If the lipid disruption we observed with HCQ in lung and kidney cells is acting through the same membrane-mediated pathway as shown for general anesthetics, then we expect that HCQ also releases the anesthetic-sensitive protein phospholipase D2 (PLD2) from GM1 clusters. Anesthetics such as xenon, chloroform, isoflurane, propofol, and diethyl ether all displace PLD2 from GM1 clusters to activate an anesthetic pathway^13,61^.

**Fig. 4:**
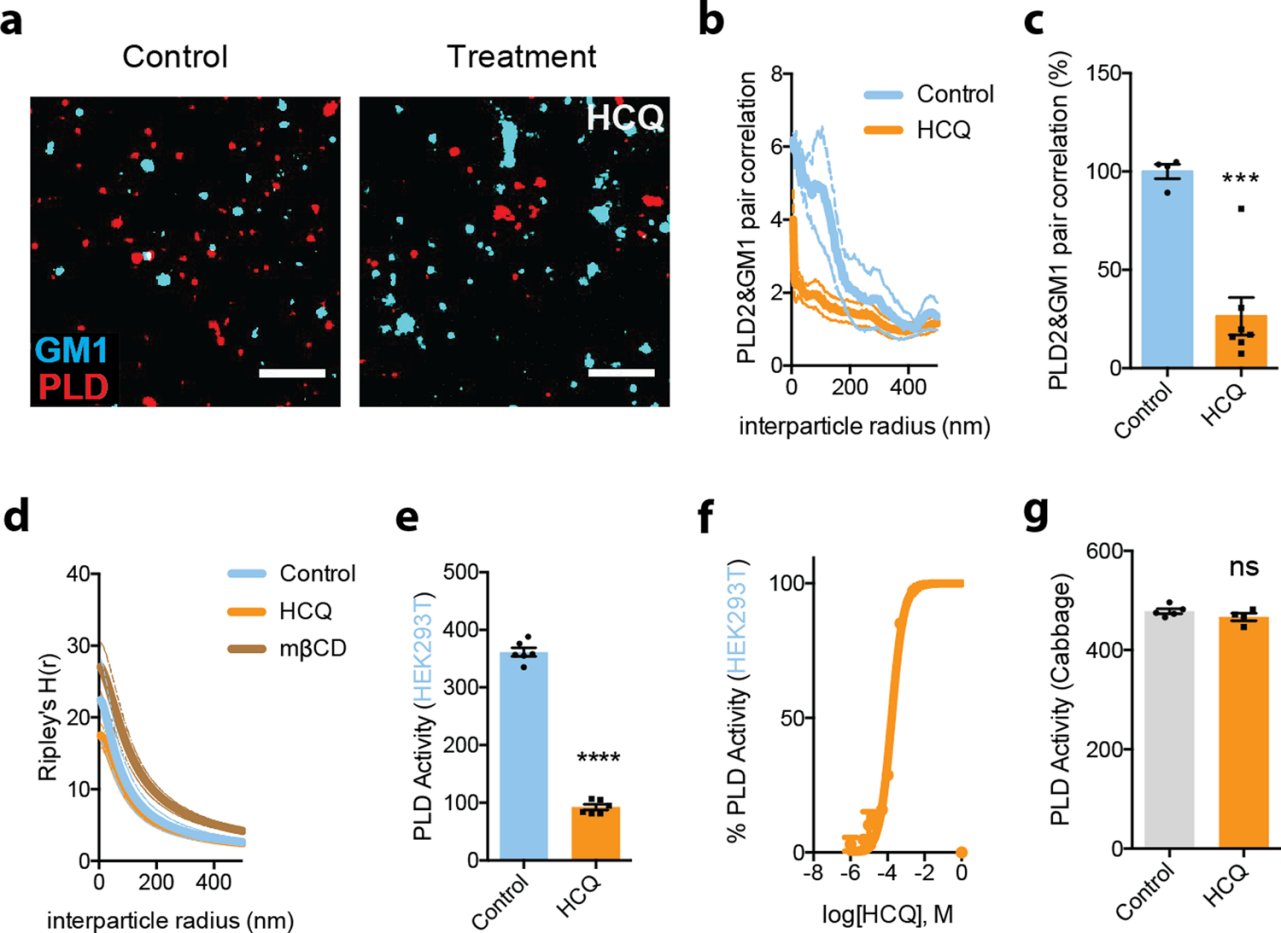
HCQ displacement of PLD2 from GM1 clusters. **a**, **b** Pair correlation analysis (**b**) of two-color dSTORM imaging (**a**). HCQ treatment decreased association of phospholipase D2 (PLD2, red shading), an anesthetic-sensitive enzyme, with GM1 cluster (cyan shading) (scale bars = 1 μm) in wt. HEK293T cells. **c** Quantification of pair correlation in **b** at short distances (0–5 nm). Data are expressed as mean ± s.e.m., ***P ≤ 0.001, unpaired *t*-test, *n* = 4–7. **d** Ripley’s H -Function (H(r)) showing cluster separation. **e** HCQ (50 μM) decreased PLD activity in PLD assay. Data are expressed as mean ± s.e.m., ****P ≤ 0.0001, unpaired *t*-test, *n* = 6. **f** A dose response of HCQ’s inhibition to PLD activity in PLD assay, *n* = 3. **g** Effect of HCQ (50 μM) on PLD activity in cabbage PLD assay is not significant. Data are expressed as mean ± s.e.m., ns not significant, unpaired *t*-test, *n* = 4–5.

To confirm HCQ’s anesthetic-like effect, clustering of PLD2 with GM1 lipids was monitored by two-color dSTORM in HEK293T cells with and without 50 µM HCQ treatment. 50 µM HCQ robustly disrupted PLD2 localization with GM1 clusters (Fig. 4a, b). Quantification of the % pair correlation at short radiuses (0–10 nm) decreased by 74 ± 13% (Fig. 4c). And this correlated with a decrease in the space between clusters (Fig. 4d). Hence HCQ’s effect on the lipid membrane is similar to general anesthetics (Supplementary Fig. 2a) in HEK293T cells. After treatment, the clusters are larger, and the ability to retain a palmitoylated protein (PLD2) is inhibited^13^.

Next, we tested the enzymatic activity of PLD2 in the presence of 50 µM HCQ. HCQ robustly inhibited PLD2 with a EC50 of 167 µM (standard error: 96–291 µM, Fig. 4e, f). The inhibition initially appeared similar to the direct binging that local anesthetic use to inhibit PLD^20^. However, when we tested HCQ’s ability to directly inhibit purified cabbage PLD, it had no effect (Fig. 4g), suggesting HCQ either has specificity for mammalian PLD2 over cabbage PLD, or HCQ inhibits by blocking its access to PIP_2_.

### Erythromycin inhibits viral entry through perturbing GM1 clusters

Azithromycin is an antibiotic derived from erythromycin that is sometimes given in combination with HCQ. Although azithromycin has shown antiviral properties in numerous studies^62–65^, the results of its usage with COVID-19 patients in combination with HCQ have been mixed^8^. Based on the cholesterol sensitivity of SARS-CoV-2, we hypothesized that erythromycin could contribute to an antiviral effect through disruption of GM1 clusters leading us test to its effects on SARS2-PV.

We found erythromycin (100 µg/mL^66^) significantly inhibits SARS2-PV infection 69 ± 17% in HEK293T cells overexpressing ACE2 at normal cholesterol levels (Supplementary Fig. 5a). Consistent with the disruptive mechanism, the same treatment increased membrane fluidity 70 ± 11% (Supplementary Fig. 5b). Furthermore, using PLD2 activity as a surrogate for an effect in live cells showed a 12 ± 4% increase (Supplementary Fig. 5c), consistent with cluster disruption. When the cholesterol level was increased using apoE and serum, erythromycin was no longer effective. In fact, when the pair correlation of ACE2 and GM1 correlation was examined, the association of ACE2 with GM1 increased by 97 ± 55% (Supplementary Fig. 3e, f). This suggests erythromycin is unable to overcome the cholesterol-induced clustering of ACE2 with GM1 lipids in elevated cholesterol.

### HCQ’s disruption of host defense peptides

Lastly, HCQ’s effects on host defense peptides were considered. Host defense peptides are amphipathic antimicrobial peptides that are upregulated during an immune response and perturb the membranes of microbes^67,68^. Cholesterol and GM1 cluster integrity show great importance to the modulation of both innate and acquired immune responses^31^. Amyloid-beta (Aβ) has been demonstrated to protect against microbial infection as a host defense peptide. The production of Aβ is regulated by the delivery of cholesterols to neurons by apoE^32^. ApoE regulates hydrolysis of amyloid precursor protein (APP) by clustering mechanism (Supplementary Fig. 1i). If HCQ disrupts GM1 lipids, then it is expected that HCQ to decrease APP pair correlation with GM1 lipids.

Aβ production was measured in HCQ-treated cells using a sandwich enzyme-linked immunosorbent assay (ELISA). HCQ was found to reduce Aβ generation by 11 ± 4% in cultured HEK293T cells (Supplementary Fig. 5d). The observed effect was statistically significant (*p* < 0.05). HEK293T cells were then loaded with cholesterol (apoE + serum) to better reflect the disease state of COVID-19 with severe symptoms. In the high-cholesterol state, HCQ did not inhibit Aβ production, resulting in a 24 ± 4% rise compared to production in low cholesterol. Since tetracaine and propofol also disrupt GM1 clusters, their effects on Aβ production were also tested and found to be very similar in both high- and low-cholesterol settings (Supplementary Fig. 5d, e).

## Discussion

**Fig. 5:**
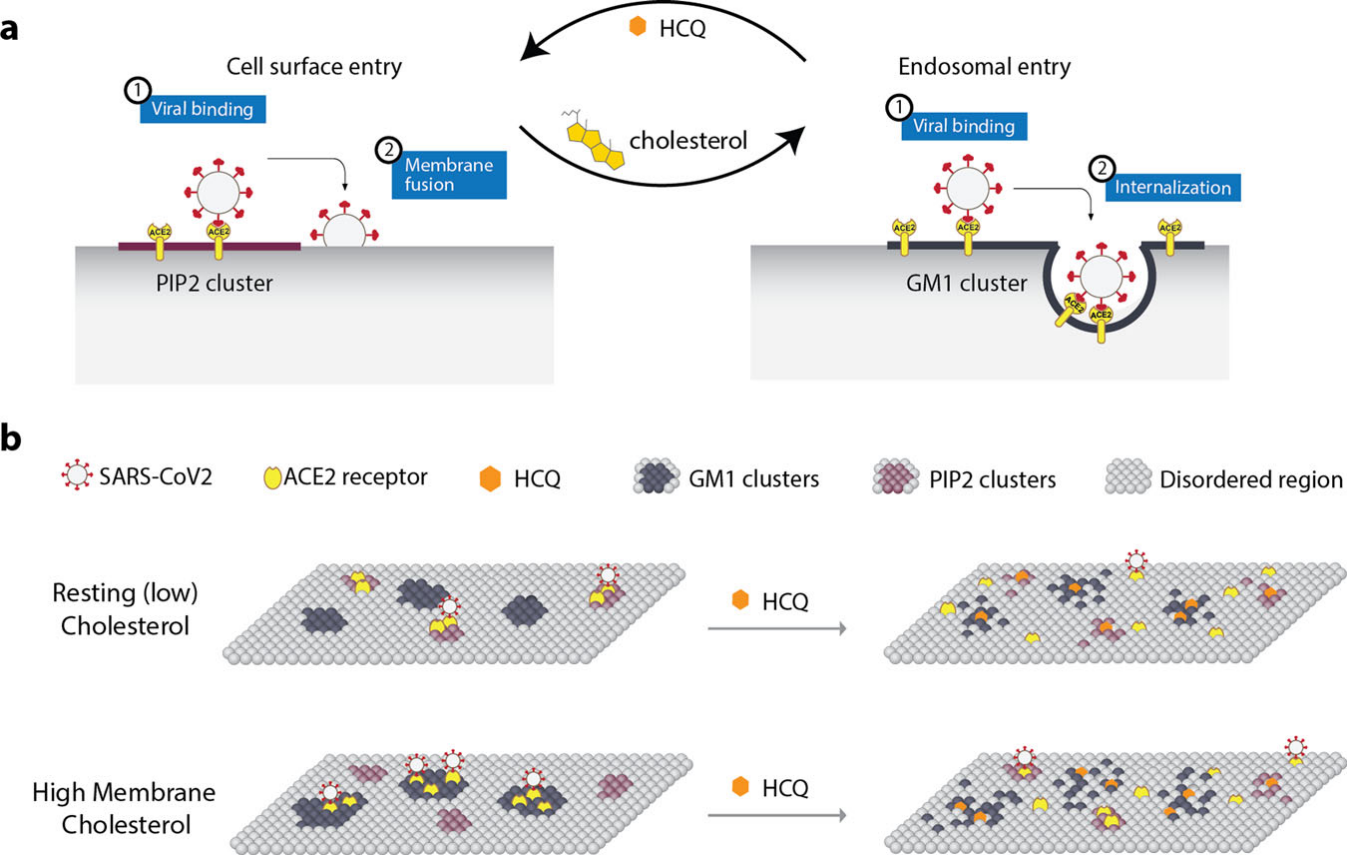
Model for HCQ mechanism of action in SARS-CoV-2 infectivity. **a** Cellular view. Cholesterol shifts viral entry from cell surface entry (at disordered regions) to endosomal entry (at GM1 clusters) and HCQ counteracts such effect. **b** Molecular view. A representation of the plasma membrane shows nanoscale lipid heterogeneity. Saturated monosialotetrahexosylganglioside1 (GM1) lipid clusters (dark grey) attract ACE2 (yellow oval) in high cholesterol (top). In low or normal cholesterol, ACE2 associates primarily with phosphatidylinositol 4,5-bisphosphate (PIP_2_). Hydroxychloroquine (HCQ, orange hexagon) disrupts the lipid order and excludes the association of ACE2 from both GM1 clusters and PIP_2_ clusters (right panel). The SARS-CoV-2 virus (white circle with red spike) binds to the ACE2 receptor. The location of the ACE2 receptor dictates the location and efficacy of viral entry.

Taken together, our findings show that HCQ has its greatest effect on cells with high cholesterol (Figs. 2i and 3g, i). In a high-cholesterol state, ACE2 is typically associated with endocytic lipids and moves primarily to disordered lipids. In a low-cholesterol state, ACE2 is associated primarily with PIP_2_ clusters and also moves to disordered lipids. The location of ACE2 in low lipids is consistent with a surface entry mechanism. Figure 5a (left panel) shows a proposed cellular model for HCQ’s membrane effect on viral entry in low cholesterol. As cholesterol increases (e.g., in COPD patients that smoke (Fig. 1e) or those with underlying conditions^28^), ACE2 shifts to endocytic lipids, and HCQ disrupts this interaction.

Previous studies have sought to address the discrepancy between in vitro and in vivo experiments with HCQ^69^. The partitioning of HCQ into negatively charged endosomes is thought to contribute^69^. PIP_2_ clusters are also highly negatively charged and likely drive the partitioning of HCQ. Elevated cholesterol is a second contributor revealed by our studies. And since HCQ opposes the effects of cholesterol, both animal and cultured-cell experiments in low cholesterol likely fail to capture the full benefits of HCQ. For example, lung cells and monkey’s studies showed no reduced effect in using HCQ in a low-cholesterol state (reflecting the physiological state of a child or healthy adult), but it does not reflect the risk an obese patient has of death from severe COVID-19 symptoms^70,71^, nor the potential of HCQ to affect ACE2 in a high-cholesterol state.

The more recent omicron variant has been shown to enter primarily through the endocytic pathway^72,73^. The omicron variant is also more infectious in children and healthy adults^74^, further supporting our findings here that moving the virus into the endocytic pathway increases infectivity. Stratifying patients by the level of lung cholesterol may reveal a benefit for HCQ and help avoid potential toxicity of HCQ in some cell types (Supplementary Fig. 1c, d). Cholesterol is typically measured in the blood, not the tissue. Blood is primarily a transient transport system and thus may not accurately predict years of accumulation in the lung of obese or chronically inflamed patients^28,31^. In conclusion, better diagnostics of cholesterol in lung tissue are needed to assess the ideal conditions for testing HCQ treatment.

Figure 5b shows a proposed molecular model of HCQ disrupting SARS-CoV-2 viral entry through perturbation of GM1 clustering. In an inflamed state, cholesterol traffics ACE2 from PIP_2_ clusters to GM1 lipids, where virions dock and enter through the endocytic pathway. The perturbation of both GM1 and PIP_2_ clusters by HCQ likely inhibits viral entry by decreasing the clustering of ACE2. The mechanism of surface receptor clustering was also shown to be important to influenza virus^54^.

ACE2 expression levels vary greatly between cell types leading us to investigate multiple types of lung and kidney cells. Furthermore, HEK293T overexpressing ACE2 is widely used in SARS-CoV-2 studies to understand how ACE2 facilitates viral entry with a stable expression of ACE2. However, the overexpression could artificially drive ACE2 clustering with GM1 lipids or artificially increase the concentration of ACE2 in the disordered region. For this reason, we used wild-type HEK293T without overexpression of ACE2 in our dSTORM experiments and compared the results to additional cell types. VeroE6 cells are highly susceptible to SARS-CoV-2 infection and used for testing drug effects on viral entry. Both A549 and H1793 are lung cell lines that mimic an early site of infection.

It is unclear where ACE2 resides when it is excluded from both GM1 clusters and PIP_2_ clusters. Presumably, it moves into a generic set of disordered lipids on the plasma membrane, but these lipids would need to be identified if they are clustered. Alternatively, ACE2 may move into PIP_3_ domains. PIP_3_ is separate from GM1 and PIP3 domains^18^.

Erythromycin contains a tertiary amine similar to HCQ. Other aminoglycosides (e.g., neomycin) are known to bind tightly to and scavenge PIP_2_. Scavenging PIP_2_ is normally thought to block ligand binding^60,75^ or change a surface charge. Our data here suggest hydrophobic-charged molecules disrupt both PIP_2_ and the resulting clustering with ACE2. Many proteins are likely affected by decreased localization to PIP_2_ clusters.

As mentioned, prior models speculated that HCQ inhibits cathepsin-L by changing the endosomal pH^46^. While we did not actually test if SARS2-PV reaches the endosome, the lack of access to endocytic lipids in the HCQ-treated cells suggests that the virus may never reach cathepsin-L, and blocking cholesterol uptake appears to inhibit viral entry^76^. Another model suggests that the HCQ could inhibit the SARS-CoV-2 viral entry step by inhibiting the binding of the spike protein to the sugar head group of GM1^77,78^. Glycosylation often affects the trafficking of proteins, and the two mechanisms could be complementary. However, it is not known if GM1 clustering or cholesterol contributes to glycosylation.

The similarities between HCQ and anesthetics in their chemical properties, viral entry inhibition, and disruption on GM1 clusters suggest that both HCQ and anesthetics could share a parallel mechanism of action. Our data suggest a shared mechanism based on chaotropic disruption of ordered lipids. Consistent with this hypothesis, CQ exhibits side effects similar to those reported in anesthetics^79^. Since HCQ partitions into the membrane, the mole fraction of the drug in the membrane is the clinically relevant concentration, not the concentration in the buffer. Based on the significant inhibition of SARS2-PV entry from tetracaine and propofol, a local anesthetic, and a general anesthetic, other anesthetic-like chemicals are likely helpful in treating COVID-19. Anesthetic with sugammadex dramatically decreases post-operative pulmonary complication^80^.

HCQ likely has a similar mechanism of action in treating Niemann-Pick syndrome, which is caused by increased GM1 clustering in the plasma membrane. HCQ likely disrupts the excess lipids in the membrane, thus reversing their effect. For example, Niemann-Pick syndrome increases APP clustering^81^, and we showed that HCQ decreases the clustering of APP (Supplementary Fig. 5d, e). The number of proteins that could be affected by clustering is extensive and warrant investigation.

HEK293T was recently shown to express low levels of endogenous ACE2^59,73^. The low levels are most similar to in vivo expression of ACE2^28^. Overexpression of ACE2 likely causes loss of GM1 regulation, and the enzyme likely distributes into both GM1 and non-GM1 regions^28^. Hence, the increased viral infection in ACE2 overexpressed cells likely includes the avidity and random association of lots of particles irrespective of ACE2 specific clustering with GM1, and the reduced efficacy of tetracaine and HCQ inhibiting viral entry in high cholesterol (57.7% vs. 50.3% and 15.6% vs. 12.8%, respectively, see Fig. 1d) may be underestimated by ACE2 overexpression. Nonetheless, in wild-type cells, ACE2 localization is still sensitive to cholesterol, and the same trend should hold true. This conclusion is further supported in Fig. S5D-E where erythromycin was unable to block Aβ production in high cholesterol.

Lastly, all the imaging was performed with endogenously expressed proteins to avoid loss of GM1 clusters associated with the regulation of ACE2. The lipids were labeled after fixing to reduce movement between clusters during labeling and to limit potential local lipid clustering by CTxB, especially saturated lipids. CTxB is pentadentate and in unfixed lipids causes clustering^82^, and to some degree, CTxB clustering occurs in fixed cells^19^. Some CTxB clustering is similar to a technique reported by Reinhard Jahn called antibody patching. This technique uses limited artificial clustering to better define the locations of protein. However, artificial clustering can affect the absolute size of a GM1 cluster, which is why we drew no significant conclusion from the absolute size of the GM1 clusters. Since we examined disruption of lipids, the CTxB could have decreased the amount of disruption we reported for apparent GM1 cluster size (i.e., the amount of disruption may be under-reported), which is why we describe an “apparent cluster” size. We do not know the absolute cluster size with certainty. PIP_2_ is polyunsaturated, and we expect it is much better fixed in the membrane. Nonetheless, we refer to apparent cluster size as an abundance of caution since this study focused on a change in cluster size and not a structural characterization of their cellular makeup.

## Methods

### Reagents

Hydroxychloroquine, PLD assay reagent amplex red 10-Acetyl-3,7-dihydroxyphenoxazine, and 2-dioctanoyl-sn-glycero-3-phosphocholine (C8-PC) were purchase from Cayman Chemical and tetracaine, methylbetacyclodextrin (MβCD), and cabbage PLD were purchased from Sigma–Aldrich. Horseradish peroxidase and choline oxidase were purchased from VWR.

### Pseudotyped SARS-CoV-2 (SARS2-PV) viral entry assay

#### Cells and virus

HEK293T cells (ATCC, CRL-3216) and HEK293T-ACE2 overexpressing cells (generated and provided by the Farzan lab^83^) were cultured in Dulbecco’s Modified Eagle Medium (DMEM, Corning^TM^, #10-013-CV) with 10% fetal bovine serum (FBS, Sigma–Aldrich, #F0926) and 1% Penicillin Streptomycin (PS, Corning^TM^, #30-002-Cl) at 37 °C with 5% CO_2_. A549 cells (ATCC, CCL-185) were cultured in F-12K Medium (Gibco, #21127-022) with 10% FBS, 1% GlutaMAX (Gibco, #35050-061), 1% Minimum Essential Medium Non-Essential Amino Acids (MEM NEAA, Gibco, #11140-050), and 1% PS, at 37 °C with 5% CO_2_. H1793 cells (ATCC, CRL-5896) were cultured in DMEM/F12 medium with 5% FBS, 1% GlutaMax, and 1% PS, at 37 °C with 5% CO_2_. Vero E6 cells (ATCC, CCL-81) were cultured in Eagle’s Minimum Essential Medium (EMEM, ATCC, 30-2003) with 10% FBS and 1% PS at 37 °C with 5% CO_2_.

SARS-CoV-2 pseudotyped particles were constructed using plasmid co-transfection, and the particles were maintained at −80 °C. The constructs were a gift from Dr. Mike Farzan, Scripps Research, Florida. Evaluation of antiviral activities in HEK293T-ACE2 overexpressing cells (0.5 × 105 cells/well), also provided by Dr. Mike Farzan, were cultured in 96-well cell-culture plates (Corning^TM^ Coastar^TM^ Cell Culture 96-well plates, #3585) and incubated with 100 μL pseudotyped particles of each type, together with 50 μM hydroxychloroquine sulfate (HCQ, Cayman, #17911) or 50 μM tetracaine hydrochloride (Sigma–Aldrich, #T7508) for 1 h. Then, the virus-drug mixture was removed, and a fresh medium was added. After 24 h, the particle yields were determined through a luciferase assay. Cells were washed with PBS, and 16 μL Cell Culture Lysis Reagent (Promega, #E153A) was added to each well. The plate was incubated for 15 min. with rocking at room temperature. Eight microliters of cell lysate from each well was added into a 384-well plate, followed by the addition of 16 μL of Luciferase Assay Substrate (Promega, #E151A). Luciferase activity measurements were performed on a Spark 20 M multimode microplate reader (Tecan). The luciferase activity, interpreted as infection, was plotted in GraphPad Prism 6 software. All the infection experiments were performed in a biosafety level-2 (BLS-2) laboratory.

### Super-resolution microscopy (dSTORM)

To detect the perturbation to GM1 clusters^14^ by hydroxychloroquine (HCQ), we employed Super Resolution Microscopy. Briefly, HEK293T cells were grown in 8-well chamber slides (Nunc Lab-Tek chamber slide system, Thermo Fisher Scientific), washed, and treated with 30–50 μM hydroxychloroquine for 30 min. The cells were then fixed with 3% paraformaldehyde, 0.1% glutaraldehyde, 30–50 μM hydroxychloroquine for 20 min, and quenched with 0.1% NaBH_4_ for 7 min. Cells were then washed with PBS (three times) and permeabilized with 0.2% TritonX 100 in PBS for 15 min. The permeabilized cells were blocked using a standard blocking buffer containing 10% BSA and 0.05% Triton in PBS for 90 min. For labeling, cells were incubated with primary antibody (anti-ACE2 antibody (Abcam, #ab189168; SinoBiological, 80031-RP01), anti-PLD2 antibody (Cell Signaling Technology, 13904 S), or anti-PIP_2_ antibody (Echelon Biosciences, Z-P045)) for 60 min in antibody buffer (PBS with 5% BSA and 0.05% TritonX-100) at room temperature followed by five washes with wash buffer (PBS with 1% BSA and 0.05% TritonX-100) for 15 min each. Secondary antibodies (Cy3B conjugated donkey anti-rabbit (Jackson Immuno Research, 711-005-152) and Alexa 647 conjugated Cholera Toxin Subunit B (CTxB, Thermo Fisher Scientific, C34778)) were added with antibody buffer for 30 min at room temperature followed by five washes as stated above. Then, cells were washed with PBS for 5 min and fixed for 10 min with fixation buffer as above, followed by 5 min washes with PBS three times, and 3 min washes with deionized distilled water. All steps except for pre- and postfixation were performed with shaking. See below for antibody validation.

A Zeiss Elyra PS1 microscope was used for super-resolution microscopy with an oil-immersed ×63 objective lens in TIRF mode. Images were acquired by Andor iXon 897 EMCCD camera and Zen 10D software with an exposure time of 18 ms per acquisition. Total 7000–10,000 frames were collected. Alexa Fluor 647 and Cy3B were excited with a 642 nm and 561 nm laser in a photo-switching buffer consisting 1% betamercaptoethanol, 0.4 mg glucose oxidase, and 23.8 µg catalase (oxygen scavengers), 50 mM Tris, 10 mM NaCl, and 10% glucose at pH 8.0. Localization drifts were corrected with n autocorrelative algorithm^84^. The drift-corrected coordinates were converted to be compatible with Vutara SRX software by an Excel macro. Cluster analysis and pair correlations were determined with the default modules in Vutara SRX software. DBSCAN algorithm was applied to determine the clusters which are within the search radius (ε) of 100 nm and consisting of at least 10 localizations. The apparent cluster size was calculated by measuring the full-width half max (FWHM) of the clusters. Pair correlation was also determined in the Vutara SRX software. The data were binned at 5 nm increments. The radial precision of our instrument is between 4 and 8 nm (Supplementary Fig. 6). We looked at the data for each curve and used either 0–5 nm or 5–10 nm as the quality of the data dictated. When calculating statistical significance by comparing the pair correlation of two conditions (e.g., with and without HCQ), the samples were always prepared and imaged at the same time. The data at the shortest binned distance (5 nm) was used unless the amount or quality of the data was insufficient, in which case 0–10 nm was used (see also the experimental setup in Fig. 2a).

### Sandwich ELISA assay

HEK293T cells were cultured in 96-well cell-culture plates. Each well was incubated with and without 100 µL treatments for 1 h, then washed with 100 µL PBS once and incubated with 100 µL PBS for 1 h. Supernatants were collected and analyzed for Aβ40 ELISA.

A 96-well plate was coated with 50 µL capture antibody (IBL #11088) at 5 µg/ml concentration in PBS and incubated overnight at 4 °C. All of the rest incubations were performed at room temperature. The plate was washed with 200 µL PBS three times, and 100 µL blocking buffer (PBS with 10%BSA and 0.05% TritonX-100) was added to each well and incubated for 1 h. Next, the blocking buffer was removed, and 50 µL of supernatant was added to each well and incubated for 1 h, followed by the addition of 50 µL primary antibody (Invitrogen^TM^ #PA3-16760) at 1:10,000 dilution in PBST buffer (PBS with 0.01% TritonX-100). After a 3 h incubation, the plate was washed with 200 µL PBST four times, and 100 µL HRP-linked goat anti-rabbit IgG secondary antibody (Invitrogen^TM^ #31460) at 0.4 µg/ml concentration in PBST buffer was added for 1 h incubation in the dark. Then, the plate was washed with 200 µL PBST four times. 80 µL Chromogen (Invitrogen^TM^ #002023) was added and incubated in the dark for 30 min. Finally, 80 µL stop solution (Invitrogen^TM^ #SS04) was applied to terminate the substrate development. Measurement of absorbance at 450 nm was performed on a microplate reader (Tecan Infinite 200 PRO) to determine relative Ab40 concentration.

### Antibody validation

The specificity of anti-ACE2 antibodies (Abcam, #ab189168, used on HEK293T cells and Vero cells; SinoBiological, 80031-RP01, used on A549 cells and H1793 cells) have been validated by western blot (Abcam) and ELISA (SinoBiological) (see manufacture product page). A549 cells have been reported to show consistent expression of ACE2^85^, and both HEK293T cells and A549 cells are susceptible to SARS-CoV-2^26^.

The specificity of the anti-PLD2 antibody (Cell Signaling Technology, 13904 S) has also been validated by western blot (see manufacture product page). For HEK293T cells, the western blot analysis of cell extracts shows no band in the wild-type. One possibility is that the wild-type cells have a significantly lower expression level compared to the PLD2 overexpressed cells, and such a difference weakens the sensitivity of this assay when most antibodies bind to the overexpression lane. This western blot analysis further validates the specificity of the antibody in HEK293T cells. Besides, HEK293T cells have been reported to show PLD activity without transfection^86^.

The specificity of anti-PIP2 antibody (Echelon Biosciences, Z-P045) has been validated by PLCδ1 competition and neomycin inhibition^87^.

### Membrane fluidity test

The change of membrane fluidity of HEK293T cells was measured using the Membrane Fluidity kit (Abcam) following the manufacturer’s protocol. Briefly, ~10,000 cells were seeded in 96-well plates and incubated with the drugs and the fluorescent lipid reagent containing pyrene decanoic acid (2 mM) at room temperature for 20–30 min. with. Pyrene decanoic acid exists as either a monomer or an excimer, the latter forms due to the change in the membrane fluidity. The formation of the excimers shifts the emission spectrum of the pyrene probe to a longer wavelength. The changes in spectrum emission were measured with a fluorescence microplate reader (Tecan Infinite 200 Pro). The ratio of monomer (EM 372 nm) to excimer (EM 470 nm) fluorescence was calculated to obtain a quantitative change in the membrane fluidity.

### In vitro cellular and enzymatic PLD assay

In vitro cellular PLD2 activity was measured in cultured HEK293T cells by an enzyme-coupled product release assay^14^ using amplex red reagent. Cells were seeded into 96-well plates (~5 × 10^4^ cells per well) and incubated at 37 °C overnight to reach confluency. The cells were starved with serum-free DMEM for a day and washed once with PBS (phosphate-buffered saline). The PLD reaction was initiated by adding 100 μL of reaction buffer (100 μM amplex red, 2 U/ml horseradish peroxidase (HRP), 0.2 U/ml choline oxidase, and 60 μM C8-PC in PBS). The assay reaction was performed for 2–4 h at 37 °C, and the activity was kinetically measured with a fluorescence microplate reader (Tecan Infinite 200 Pro) at excitation and emission wavelengths of 530 nm and 585 nm, respectively. For enzymatic PLD assay, cabbage PLD was used instead of the live cells, and the PLD reaction was initiated as described for the cellular assay. The PLD2 activity was calculated by subtracting the background activity (reaction buffer with the drugs, but no cells). For the bar graphs, samples were normalized to the control activity at the 120 min time point.

### In vitro free-cholesterol assay

In vitro free-cholesterol assay was measured in lysed human lung tissue, A549 cells, and H1793 cells by an Amplex Red-based assay^28^. Airway tissue samples (~1 mg) were fixed in PFA and stored at 4 degrees prior to the assay. Cells were cultured with the complete medium as described earlier in 96-well plates until ~90% confluent and fixed with PFA. Lung tissue and cultured cells were homogenized with RIPA lysis buffer (Thermo Fisher Scientific 89900) overnight and centrifuged to collect supernatant for cholesterol and protein measurement. Protein concentration was determined through NanoDrop A280 measurement. Samples normalized by protein concentration were mixed with assay buffer containing 100 μM Amplex red, 2 U/mL horseradish peroxidase, 4 U/mL cholesterol oxidase, and 4 U/mL cholesteryl esterase in PBS to reach 100 μL of reaction volume. The assay reaction was measured by a fluorescence microplate reader (Tecan Infinite 200 PRO) at an excitation wavelength of 530 nm and an emission wavelength of 585 nm. Relative cholesterol concentration was determined by fluorescence activity and calculated by subtracting the background activity of the reaction buffer without samples.

### Fixable viability dye cell staining

Fixable Viability Dye (FVD) eFluor^TM^ 506 (Invitrogen, 65-0866) was used to label dead cells in both HEK293T cell and Vero E6 cell cultures with or without HCQ treatment. Cells were cultured as previously described in 96-well plates until ~90% confluent and treated with or without 50 μM HCQ for 1 h before the staining. Cells were scraped off by 1000 μL pipette tips and transferred to V-bottom 96-well plates (Corning 3894). Next, cells were washed with 200 μL PBS twice and incubated with 100 μL FVD (1:1000) for 30 min at 4 °C in dark. After that, cells were washed with 200 μL PBS twice and analyzed by MACSQuant^®^ Analyzer 10 Flow Cytometer. Data were analyzed by FlowJo.

### MTT assay

MTT assay kit (Abcam, Ab211091) was applied to assess cell metabolic activity in both HEK293T cell and Vero E6 cell cultures with or without HCQ/tetracaine/propofol treatment. Cells were cultured as previously described in 96-well plates until ~90% confluent and treated with or without 50 μM HCQ/tetracaine/propofol for 1 h before the assay. After the treatments, the cells were washed with 200 μL PBS once and incubated with 50 μL serum-free media and 50 μL MTT Reagent in each well at 37 °C for 3 h. Next, 150 μL MTT Solvent was added to each well, and the plate was shaken on an orbital shaker for 15 min in dark. Absorbance was read at OD = 590 nm by Tecan infinite M200 PRO.

### Statistics and reproducibility

Data calculations and graphs were performed in Prism 6 (GraphPad software) or Microsoft Excel. Experiments were done two-three times to ensure reproducibility. All Experimental samples were performed in random order when to avoid any experimental bias. To ensure the reproducible effect of the sample sizes, super-resolution imaging was carried out on multiple cells. Each image is a separate cell, and each ‘n’ comes from a separate image, so each n is a biological repeat. Furthermore, the data are from at least two separate experiments. Statistical significance was evaluated using one-way ANOVA with post hoc Tukey’s test, two-tailed *t*-tests, parametric or nonparametric wherever appropriate. Data are shown as the mean and the error bars with SD. Significance is indicated by ns not significant, *P ≤ 0.05, **P ≤ 0.01, ***P ≤ 0.001, and ****P ≤ 0.0001.

We have indicated the technical repeats (‘n’) for each analysis. For dSTORM imaging, the data are from at least two separate experiments. Each image is a separate cell, and each ‘n’ comes from a separate image. So, the biological repeats are the same as the technical repeats. We have indicated this in the methods describing the dSTORM. For cellular assays, each ‘n’ comes from a separate well.

### Reporting summary

Further information on research design is available in the Nature Research Reporting Summary linked to this article.

## Supplementary information

Supplementary Information

Reporting Summary

## Data Availability

Data are deposited in Mendeley Data (10.17632/swh2crwhpg.1).
