# Author Correction: Hydroxychloroquine blocks SARS-CoV-2 entry into the endocytic pathway in mammalian cell culture

**DOI:** 10.1038/s42003-022-04172-4

**Published:** 2022-11-02

**Authors:** Zixuan Yuan, Mahmud Arif Pavel, Hao Wang, Jerome C. Kwachukwu, Sonia Mediouni, Joseph Anthony Jablonski, Kendall W. Nettles, Chakravarthy B. Reddy, Susana T. Valente, Scott B. Hansen

**Affiliations:** 1 Department of Molecular Medicine, The Scripps Research Institute, Jupiter, FL 33458 USA; 2 Department of Neuroscience, The Scripps Research Institute, Jupiter, FL 33458 USA; 3 Skaggs Graduate School of Chemical and Biological Sciences, The Scripps Research Institute, Jupiter, FL 33458 USA; 4 Department of Integrative Structural and Computational Biology, The Scripps Research Institute, Jupiter, FL 33458 USA; 5 Department of Immunology and Microbiology, The Scripps Research Institute, Jupiter, FL 33458 USA; 6 Department of Internal Medicine, University of Utah Health Sciences Center, Salt Lake City, UT 84112 USA

**Keywords:** SARS-CoV-2, Nanoscale biophysics, Membrane trafficking, Membrane lipids, Sterols

Correction to: *Communications Biology* 10.1038/s42003-022-03841-8, published online 14 September 2022.

In the original version of the Article, the 8th paragraph of the discussion stated “Another model suggests that HCQ could inhibit the SARS-COV-2 viral entry step by changing the glycosylation of membrane proteins^77,78^.”

The text should read “Another model suggests that the HCQ could inhibit the SARS-CoV-2 viral entry step by inhibiting the binding of the spike protein to the sugar head group of GM1^77,78^.”

This has now been corrected in the PDF and HTML versions of the Article.

