# Supplementary material for: Hydroxychloroquine blocks SARS-CoV-2 entry into the endocytic pathway in mammalian cell culture
Source: Commun Biol. 2022 Sep 14;5:958. doi: 10.1038/s42003-022-03841-8 (PMC9472185; doi:10.1038/s42003-022-03841-8)
Supplement: Supplementary file 1 — Supplementary Information [file 42003_2022_3841_MOESM1_ESM.pdf]

Supplemental Figures

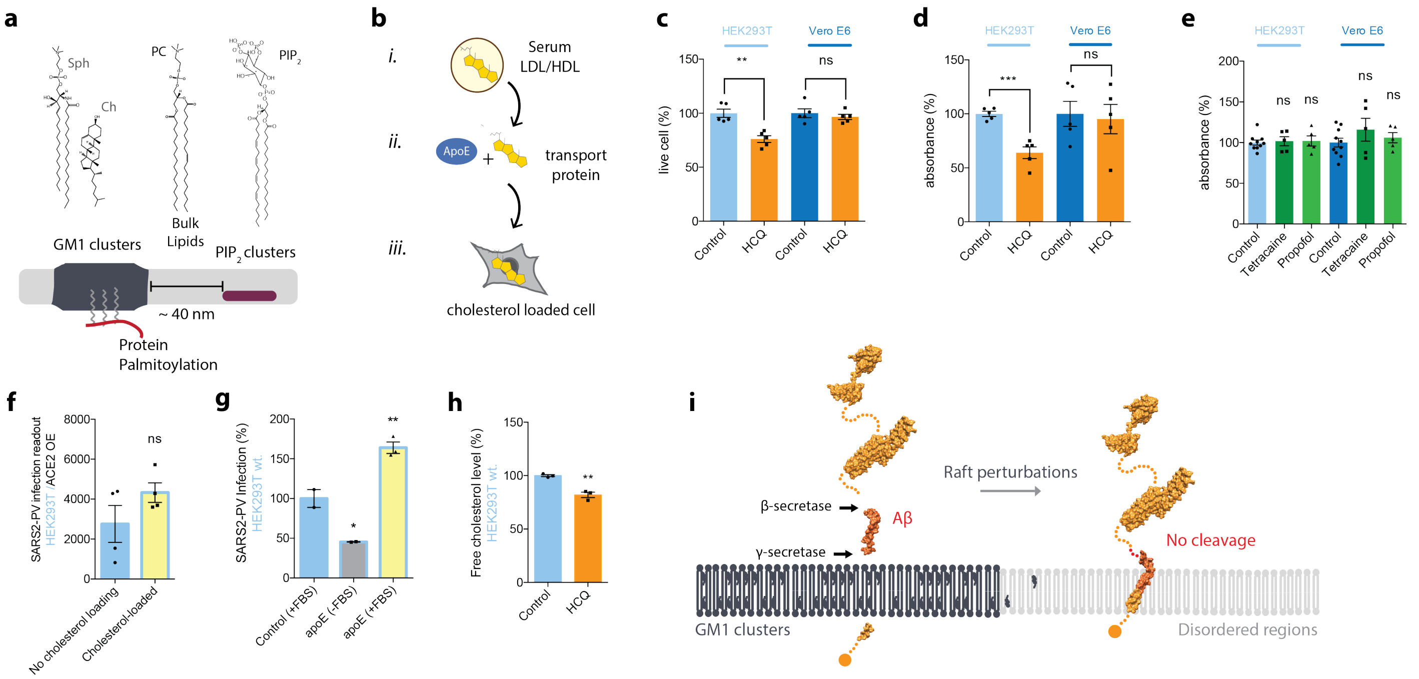

Supplementary Fig. 1. Membrane heterogeneity. a GM1 clusters are clusters of saturated known as liquid ordered (L_o) and commonly reside separate from liquid disordered (L_d) phases[19]. The ordered phase (L_o) is generally enriched in sphingomyelin and cholesterol whereas the disordered (L_d) phase consists of unsaturated lipids and includes polyunsaturated lipids like PA and PIP_2[86]. **b** Cartoon diagram showing the experimental setup for loading cultured cells with cholesterol. ***i.***, Cholesterol (yellow shading) loaded into lipoprotein (e.g., low- and high-density lipoprotein (LDL and HDL respectively)) from blood serum. ***ii.***, Cholesterol free human apolipoprotein E (apoE, brown shading), a cholesterol transport protein, is exposed to cholesterol from blood serum and ***iii,*** ApoE transports cholesterol into of cells (grey shading). **c** Live cell ratio detected by flow cytometry with Fixable Viability Dye staining of 1 h 50μM HCQ treatment on HEK293T cells and Vero E6 cells. Data are expressed as mean ± s.e.m., **P≤0.01, unpaired t-test, n=5. **d** MTT assay of 1 h 50 μM HCQ treatment on HEK293T cells and Vero E6 cells. Data are expressed as mean ± s.e.m., ***P≤0.001, unpaired t-test, n=5. **e** MTT assay of 1 h 50μM tetracaine/propofol treatment on HEK293T cells and Vero E6 cells. Data are expressed as mean ± s.e.m., unpaired t-test, n=5. **f** SARS2-PV entry in ACE2 overexpressing HEK293T cells without and with cholesterol loading indicated by raw luciferase activity readout. Data are expressed as mean ± s.e.m., unpaired t-test, n=4. **g** SARS-PV entry in wt. HEK293T cells with ApoE extracting (no FBS condition) and loading (FBS condition) cholesterol[28]. Data are expressed as mean ± s.e.m., one-way ANOVA with post hoc Tukey's test, n=2-3. **h** HCQ decreased free-cholesterol level in wt. HEK293T cells. Data are expressed as mean ± s.e.m., **P ≤ 0.01, unpaired t test, n=3. **i** Model of HCQ and anesthetics translocating APP from GM1 clusters to disordered region through cluster perturbation to reduce the synthesis of Aβ.

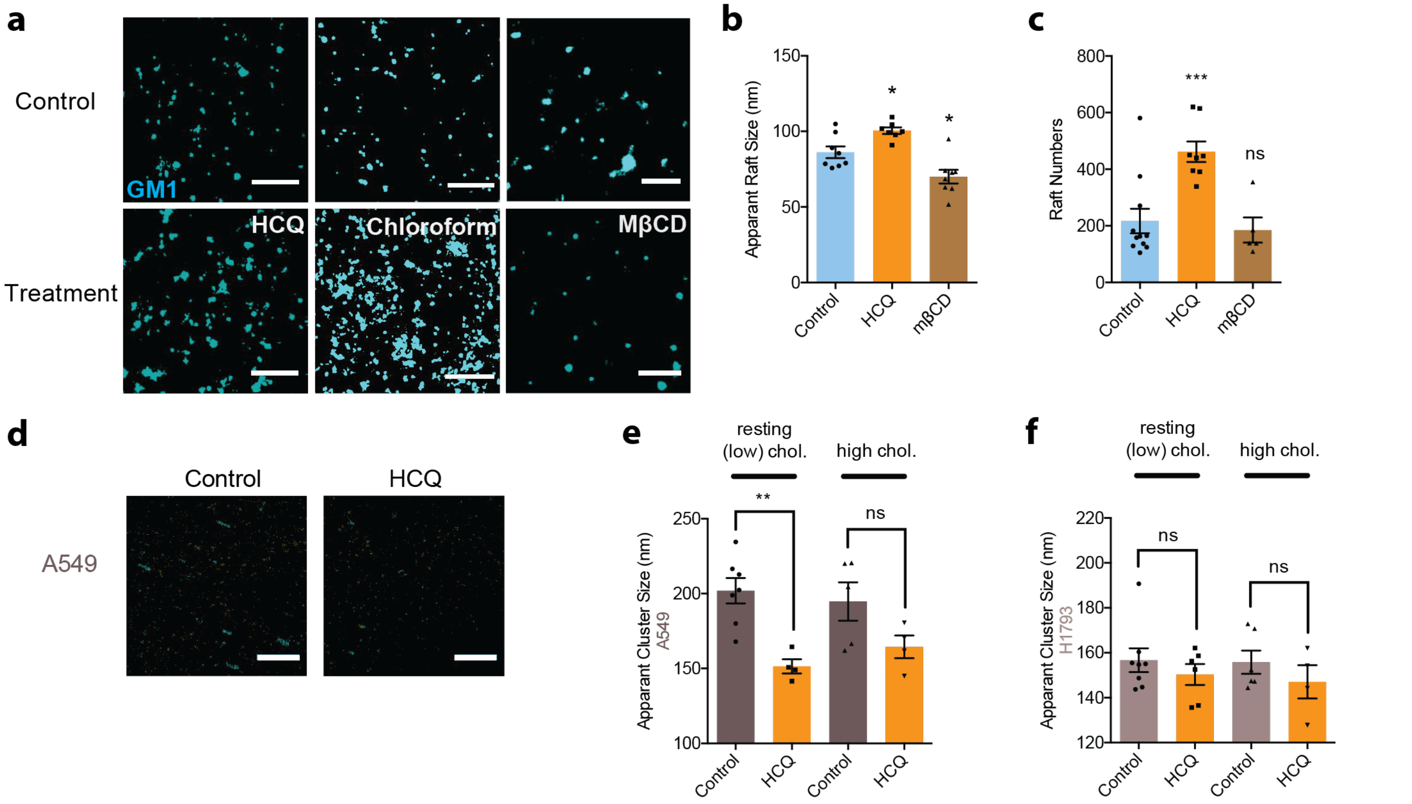

Supplementary Fig. 2. Anesthetic-like disruption of GM1 clusters by Hydroxychloroquine. a Representative dSTORM images showing the GM1 cluster perturbation by HCQ (50 µM) and MβCD (100 µM) in HEK293T cells (Scale bars = 1 µm). Similar disruption from 1 mM chloroform treatment, an anesthetic, is shown from Pavel et. al. PNAS 2020; 117:13757–66, with permission (CC BY-NC-ND 4.0), for comparison. **b-c** Bar graph of the apparent cluster diameter analyzed by DBSCAN cluster analysis. HCQ increases both cluster diameter (b) and number (c) of GM1 clusters. Data are expressed as mean ± s.e.m., *P ≤ 0.05, ***P ≤ 0.001, one-way ANOVA with post hoc Tukey's test, (n=7). **d** Representative dSTORM images showing HCQ's disruption on GM1 clusters (blue) in A549 cells. **e-f** Apparent cluster size of A549 cells (e) and H1793 cells (f) quantified by cluster analysis. Data are expressed as mean ± s.e.m., unpaired t test, n=4-8.

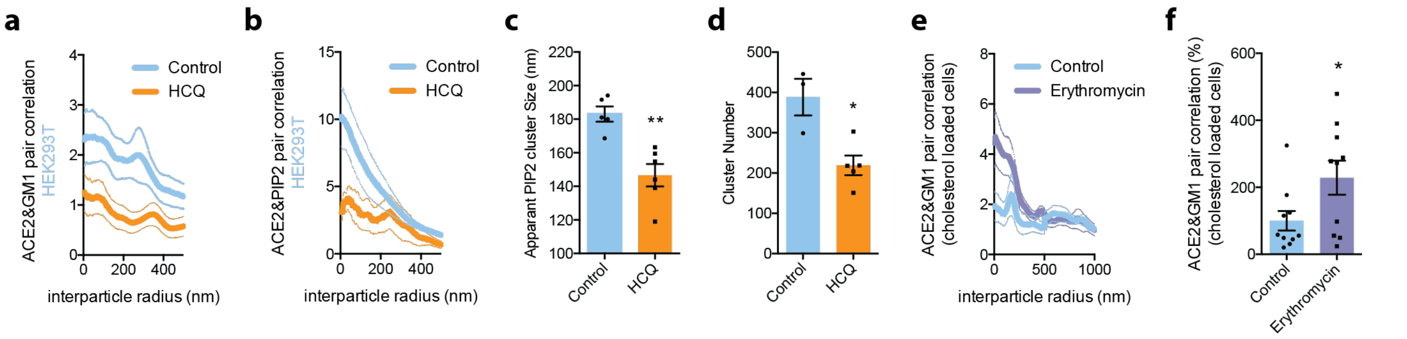

Supplementary Fig. 3. dSTORM of HEK293T cells. a-b Pair correlation analysis of dSTORM imaging in WT HEK293T (Fig. 2c, e). HCQ treatment decreased association of ACE2 with GM1 and PIP_2. **c-d** Bar graph of the apparent cluster diameter analyzed by DBSCAN cluster analysis. HCQ decreases both cluster diameter (c) and number (d) of PIP_2 clusters. Data are expressed as mean ± s.e.m., *P ≤ 0.05, **P ≤ 0.01, one-way ANOVA with post hoc Tukey's test, n=5-6. **e-f** Pair correlation (e) and percent of pair correlation calculated at short distances (0-5 nm) (f) of dSTORM imaging. Erythromycin treatment decreased association of ACE2 with GM1 clusters. Data are expressed as mean ± s.e.m., *P ≤ 0.05, **P ≤ 0.01, unpaired t test, n=10.

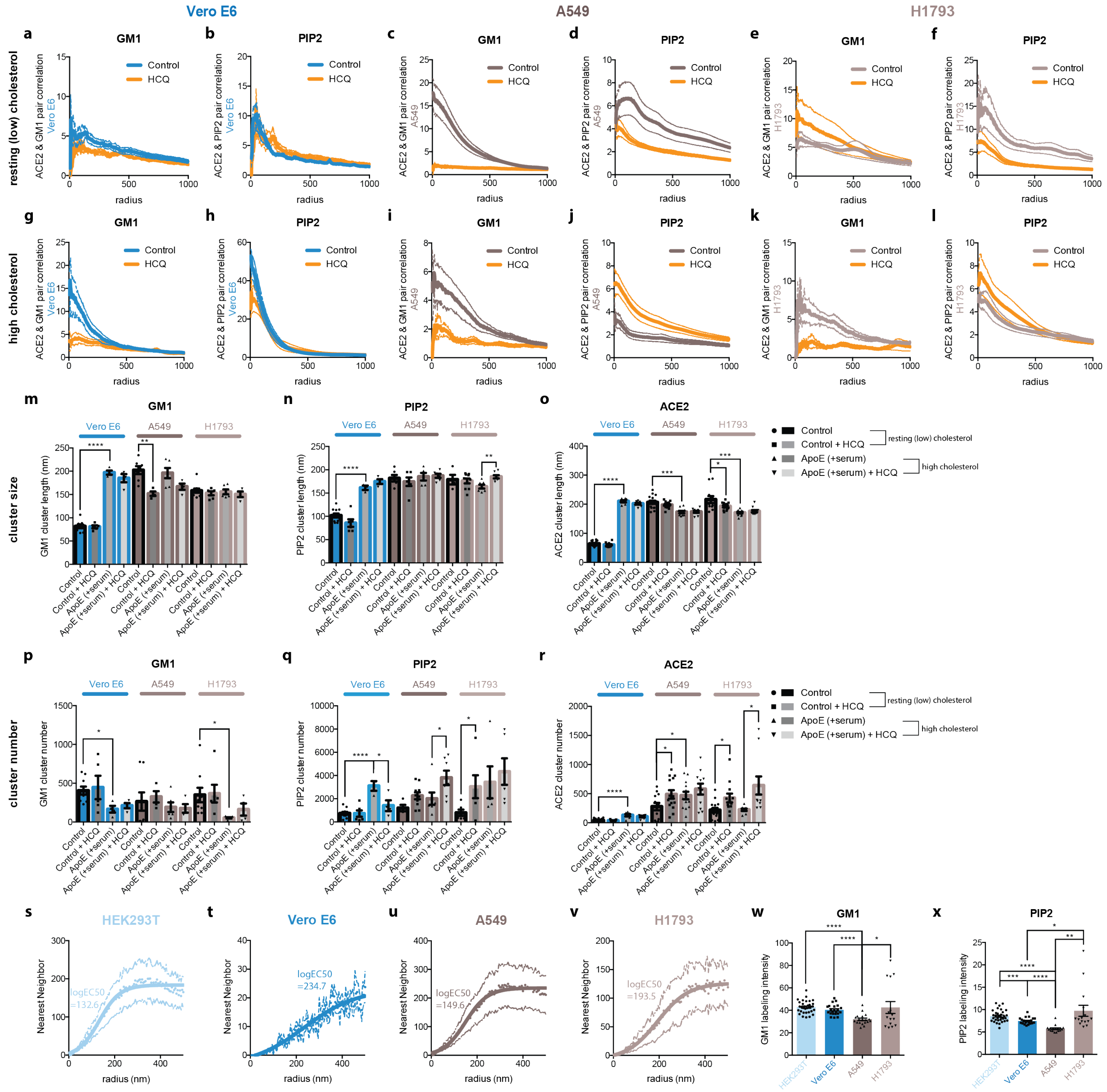

Supplementary Fig. 4. dSTORM of Vero E6, A549, and H1793 cells. a-l Raw data used to calculate the pair correlation of ACE2 with lipids. The mathematical minimum of pair correlation asymptotes at 1. Therefore, a value of 3 is 2x background and <3 is considered no significant association. Values between 3-5 are very weak but could have small amounts of localization. Values above >10 are considered strong pair correlation. The pair correlation is a unitless number. **m-o** Cluster size analysis of GM1 clusters, PIP_2 clusters, and ACE2 clusters in Vero E6, A549, and H1793 cells. **p-r** Cluster number analysis of GM1 clusters, PIP_2 clusters, and ACE2 clusters in Vero E6, A549, and H1793 cells. **s-v** Average distance (nm) of GM1 clusters and PIP_2 clusters separation is calculated through Nearest Neighbor analysis in HEK293T, Vero E6, A549, and H1793 cells. **w-x** The amount of GM1 clusters and PIP_2 clusters is examined by labeling intensity in confocal imaging (laser voltage was 750V for both probes) in HEK293T, Vero E6, A549, and H1793 cells.

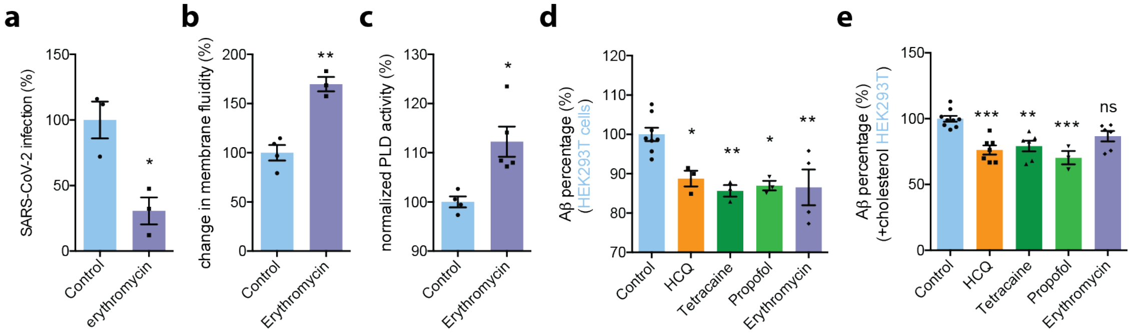

Supplementary Fig. 5. Erythromycin inhibits SARS-CoV-2 viral entry. a Percent SARS-CoV-2 pseudovirus (SARS2-PV) infection after erythromycin (100 μg/ml) treatment of HEK293T cells over expressing ACE2. Data are expressed as mean ± s.e.m., *P ≤ 0.05, unpaired t test, n=3. **b** Erythromycin (100 μg/ml) increased membrane fluidity in membrane fluidity assay. Data are expressed as mean ± s.e.m., **P ≤ 0.01, unpaired t test, n=3-4. **c** A cluster disruption assay based on PLD2 enzymatic activity in HEK293T cells. **d-e** An ELISA assay showing HCQ (50 μM), tetracaine (50 μM), propofol (100 μM), and erythromycin (100 μg/ml) decreased the synthesis of Aβ40 in HEK293T cells with (e) and without (d) cholesterol loading (4 μM apolipoprotein E + 10% serum). Data are expressed as mean ± s.e.m., *P ≤ 0.05, **P ≤ 0.01, one-way ANOVA with post hoc Tukey's test, n=3-7.

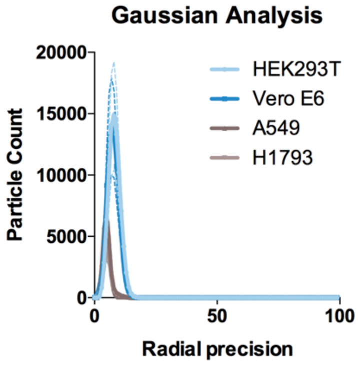

Supplementary Fig. 6. Gaussian analysis of radial precision of different cell lines. The exact values of the precision for each cell type are as follows: A549, 4.8 ± 1.4 nm; H1793, 5.1 ± 1.3 nm; VeroE6, 7.4 ± 2.4 nm; and HEK293T, 8.0 ± 2.35 nm (mean ± SD).

Cell Lines			Vero	A549	H1793	HEK
	Cholesterol	HCQ				
GM1 & ACE2 Pair Corr.	High	-	high	low	low	med
		+	low	none	none	none
	Low	-	high	high	low	-
		+	high	none	low	-
GM1 & PIP2 Pair Corr.	High	-	high	none	low	high
		+	high	low	low	low
	Low	-	high	low	med	-
		+	low	low	low	-
GM1 Cluster length (nm)	High	-	197	196	156	86.2
		+	185	167	150	100
	Low	-	81.8	240	158	-
		+	80.7	152	152	-
ACE2 cluster Length (nm)	High	-	210	171	173	-
		+	202	176	174	-
	Low	-	64.3	236	207	-
		+	60.6	193	198	-
GM1/PIP2 separation (nm)	Low	-	235	150	193	133

Supplementary Table 1. Hydroxychloroquine is show in orange. Pair correlation <3 (none), 4-6 (low), 6-10 (med), >10 (high). Low cholesterol (Chol.) is depleted, High Chol. is loaded. See Fig. S4 for representative pair correlation data.
