# Supplementary material for: Hydroxychloroquine blocks SARS-CoV-2 entry into the endocytic pathway in mammalian cell culture
Source: Commun Biol. 2022 Sep 14;5:958. doi: 10.1038/s42003-022-03841-8 (PMC9472185; doi:10.1038/s42003-022-03841-8)
Supplement: Supplementary file 2 — Reporting Summary [file 42003_2022_3841_MOESM2_ESM.pdf]

nature research

Corresponding author(s): Scott Hansen

Last updated by author(s): Jun 14, 2022

Reporting Summary

Nature Research wishes to improve the reproducibility of the work that we publish. This form provides structure for consistency and transparency in reporting. For further information on Nature Research policies, see our Editorial Policies and the Editorial Policy Checklist.

Statistics

For all statistical analyses, confirm that the following items are present in the figure legend, table legend, main text, or Methods section.

n/a	Confirmed	
☐	☒	The exact sample size (n) for each experimental group/condition, given as a discrete number and unit of measurement
☐	☒	A statement on whether measurements were taken from distinct samples or whether the same sample was measured repeatedly
☐	☒	The statistical test(s) used AND whether they are one- or two-sided<br>*Only common tests should be described solely by name; describe more complex techniques in the Methods section.*
☐	☒	A description of all covariates tested
☐	☒	A description of any assumptions or corrections, such as tests of normality and adjustment for multiple comparisons
☐	☒	A full description of the statistical parameters including central tendency (e.g. means) or other basic estimates (e.g. regression coefficient) AND variation (e.g. standard deviation) or associated estimates of uncertainty (e.g. confidence intervals)
☐	☒	For null hypothesis testing, the test statistic (e.g. F, t, r) with confidence intervals, effect sizes, degrees of freedom and P value noted<br>*Give P values as exact values whenever suitable.*
☒	☐	For Bayesian analysis, information on the choice of priors and Markov chain Monte Carlo settings
☒	☐	For hierarchical and complex designs, identification of the appropriate level for tests and full reporting of outcomes
☒	☐	Estimates of effect sizes (e.g. Cohen's d, Pearson's r), indicating how they were calculated

Our web collection on statistics for biologists contains articles on many of the points above.

Software and code

Policy information about availability of computer code

Data collection	Vutara software for dSTORM imager.
Data analysis	Vutara DBSCAN and cluster analysis software are described in the methods.

For manuscripts utilizing custom algorithms or software that are central to the research but not yet described in published literature, software must be made available to editors and reviewers. We strongly encourage code deposition in a community repository (e.g. GitHub). See the Nature Research guidelines for submitting code & software for further information.

Data

Policy information about availability of data

All manuscripts must include a data availability statement. This statement should provide the following information, where applicable:

- Accession codes, unique identifiers, or web links for publicly available datasets
- A list of figures that have associated raw data
- A description of any restrictions on data availability

Data are deposited to Mendeley Data (DOI: 10.17632/swh2crwhpg.1).

April 2020

Field-specific reporting

Please select the one below that is the best fit for your research. If you are not sure, read the appropriate sections before making your selection.

☒ Life sciences ☐ Behavioural & social sciences ☐ Ecological, evolutionary & environmental sciences

For a reference copy of the document with all sections, see nature.com/documents/nr-reporting-summary-flat.pdf

Life sciences study design

All studies must disclose on these points even when the disclosure is negative.

Sample size	For dSTORM data, each 'n' is a separate cell, one image per cell. So biological replicates are the same as technical replicates. Sample size was calculated to be between 7-8 replicates using power analysis from previous studies. Conclusions were drawn from statistically significant data. When needed the sample size was increased to 10-12.
Data exclusions	No data was excluded.
Replication	All experiments are replicates of at least two separate experiments.
Randomization	The order of imaging and treatment were randomized to avoid experimental bias.
Blinding	Samples were imaged by well number and later paired with the treatment type to avoid bias.

Reporting for specific materials, systems and methods

We require information from authors about some types of materials, experimental systems and methods used in many studies. Here, indicate whether each material, system or method listed is relevant to your study. If you are not sure if a list item applies to your research, read the appropriate section before selecting a response.

Materials & experimental systems

n/a	Involved in the study	
☐	☒	Antibodies
☐	☒	Eukaryotic cell lines
☒	☐	Palaeontology and archaeology
☒	☐	Animals and other organisms
☐	☒	Human research participants
☒	☐	Clinical data
☒	☐	Dual use research of concern

Methods

n/a	Involved in the study	
☒	☐	ChIP-seq
☒	☐	Flow cytometry
☒	☐	MRI-based neuroimaging

Antibodies

Antibodies used	Abcam, #ab189168;SinoBiological, 80031-RP01; Cell Signaling Technology, 13904S, Echelon Biosciences, Z-P045.
Validation	The specificity of anti-ACE2 antibodies (Abcam, #ab189168, used on HEK293T cells and Vero cells; SinoBiological, 80031-RP01, used on A549 cells and H1793 cells) have been validated by western blot (Abcam) and ELISA (SinoBiological) (see manufacture product page). A549 cells has been reported to show consistent expression of ACE2 [79] and both HEK293T cells and A549 cells are susceptible to SARS-CoV-2[26].<br><br>The specificity of anti-PLD2 antibody (Cell Signaling Technology, 13904S) has also been validated by western blot (see manufacture product page). For HEK293T cells, the western blot analysis of cell extracts shows no band in the wildtype. One possibility is that the wildtype cells have a significantly lower expression level compared to the PLD2 overexpressed cells and such difference weakens the sensitivity of this assay when most antibodies bind to the overexpression lane. This western blot analysis further validates the specificity of the antibody in HEK293T cells. Besides, HEK293T cells have been reported to show PLD activity without transfection[80].<br><br>The specificity of anti-PIP2 antibody (Echelon Biosciences, Z-P045) has been validated by PLC[illegible]1 competition and neomycin inhibition[81].

Eukaryotic cell lines

Policy information about cell lines

Cell line source(s)	HEK293, VeroE6, a549, H1793

Authentication	HEK293 are from ATCC.
Mycoplasma contamination	Not confirmed
Commonly misidentified lines (See ICLAC register)	none

Human research participants

Policy information about studies involving human research participants

Population characteristics	Patients from a COPD clinic at the University of Utah
Recruitment	Patients diagnosed with and without COPD were recruited from a COPD clinic.
Ethics oversight	University of Utah Hospital

Note that full information on the approval of the study protocol must also be provided in the manuscript.
